# The Perception of Consumer Behaviors in Subscription Platforms for Surplus Food Restaurants—An Analytical View of the Technology Acceptance Model

**DOI:** 10.3390/foods13193045

**Published:** 2024-09-25

**Authors:** Chun-Chieh Ma, Hsiao-Ping Chang

**Affiliations:** 1 Department of Public Administration and Management, National University of Tainan, Taiwan, No. 33, Sec. 2, Shu-Lin St., Tainan 70005, Taiwan; ccma@mail.nutn.edu.tw; 2 Department of Health Industry Technology Management, Chung Shan Medical University, No. 110, Sec. 1, Jianguo N. Rd., Taichung City 40201, Taiwan; 3 Department of Medical Management, Chung Shan Medical University Hospital, No. 110, Sec. 1, Jianguo N. Rd., Taichung City 40201, Taiwan

**Keywords:** subscription service, food waste, technology acceptance model, new environmental paradigm, environmental behavior

## Abstract

Subscription services have become popular in recent years, breaking the traditional business model of one-time payment and prompting operators to build long-term loyal relationships with their customers. As smartphones are popular in Taiwan and the Taiwanese have a high acceptance of new technologies, is it possible for domestic restaurants to reach a win-win situation for both consumers and restaurant operators and to reduce food waste through subscription services? The Technology Acceptance Model was used in this study to explore consumers’ perceived usefulness, perceived ease of use, and attitudes toward restaurant subscription platforms, with two variables, new environmental paradigm and environmental behavior, added to probe the relations with intention to use. This study was conducted by convenience and snowball sampling, and the subjects were consumers eating out. A total of 400 questionnaires were collected and 369 valid ones were returned, with a response rate of 92.25%. The results show that perceived usefulness, perceived ease of use, new environmental paradigm, environmental behaviors, and attitude toward using have significant positive effects, and attitude toward using has the same effect on intention to use. In addition, attitude toward using has a mediating effect on perceived usefulness, new environmental paradigm, environmental behavior, and intention to use. Finally, it is expected that the results of this study can be used as a reference for restaurant operators to adopt subscription services in order to build long-term and stable relationships with consumers. Furthermore, new entrepreneurs can also evaluate the feasibility of building a subscription platform like this one, which can provide a convenient and economical option for consumers dining out, as well as reduce food waste.

## 1. Introduction

With changes in today’s social structure and lifestyle, the number of consumers eating out is rising. The growing proportion of such a group over the past decade caused food waste in restaurants to increase with the diffusion of information in social networks. As the world has been impacted by COVID-19 in the past two years, consumers’ purchasing behaviors reflect that they pay more attention to the shopping experience. In addition to the traditional cost–performance ratio, consumers also hope to take into account the diversified added value. Subscription services can not only provide consumers with a variety of dining services but also play a role in solving the problem of surplus food.

Past experience revealed that fierce competition in the marketplace can force restaurant operators to diversify their menu offerings at the expense of food waste [1]. However, while 1.3 billion tons of produced food are discarded worldwide, 1 billion people are hungry at the same time [2]. Therefore, reducing food waste in restaurants means more than social and moral responsibilities, it can also be environmentally friendly [3] and improve economic efficiency [4].

In the context of escalating global food waste issues, surplus food restaurants in Taiwan offer a novel solution aimed at reducing food waste and promoting resource reutilization. Compliance with the Food Safety and Sanitation Management Act is essential, ensuring that food safety and quality are rigorously controlled through proper storage, handling procedures, and comprehensive monitoring systems [5,6]. Furthermore, the proposal for surplus food redistribution requires a well-defined operational plan that encompasses collection, storage, handling, and distribution procedures to ensure food safety and fairness [7,8]. For these reasons, using surplus food necessitates stringent hygiene practices to prevent contamination and ensure safety. According to Taiwan’s Food Safety and Sanitation Management Act, proper storage conditions and cross-contamination prevention are crucial [6]. Additionally, the Taiwanese government has implemented policies to reduce plastic usage, highlighting the importance of addressing environmental concerns [9]. Those considerations implemented in Taiwan aim to provide operational guidelines of surplus food restaurants in Taiwan and offer practical specifications for improving food resource utilization and sustainability.

Subscription services focus on customer experience and long-term value, changing the focus from corporate (product) to customer (service). In addition to customization, they are also important to improve customer stickiness to enterprises’ products or services and build a long-term relationship with customers [10]. In Taiwan, eating out has become more and more common and widely accepted by locals, and the restaurant industry has entered the era of e-commerce. Considering these facts and the high acceptance of new technology among Taiwanese consumers, it is worthwhile to further understand and explore whether domestic restaurants also adopt subscription systems. Furthermore, new entrepreneurs developing their restaurant subscription platforms may help to achieve mutual benefits for both consumers and restaurant operators while reducing food waste [3].

The Technology Acceptance Model (TAM) is commonly used to explore the development of users and the use of information technology [11]. It is also a theoretical model to help explain and predict the acceptance of users in using certain information technology and its influencing factors. It has been proven to be a theoretical model with high-quality and high-reliability statistical results in many empirical studies [12,13]. There are still many studies that examine consumers’ intention to use new technologies by TAM, most of which are in the field of e-commerce applications [14,15,16,17]. Based on the above statements and the possible impact of perceived usefulness and perceived ease of use regarding technological products or technologies on consumers’ attitudes toward use [18,19], this study aims to probe the key factors that influence consumers’ intention to use restaurant subscription platforms through the TAM.

Moreover, past findings found significant differences between the predictors of reduction, reuse, and recycling of food waste. Reduction and reuse were predicted by potential environmental values, knowledge, and concern-based variables, while recycling was described as a highly normative behavior [20]. The New Environmental Paradigm Scale is a measurement tool with a high stability used to examine people’s attitudes toward the environment [21,22,23]. Environmental behavior is a method to tackle environmental problems through strategies [24]. In this study, environmental behaviors were included in the TAM to observe the relationship between users’ environmental behaviors and their attitudes toward and intention to use restaurant subscription platforms, as environmental behaviors were related to reduction in food waste and disposal of surplus food in restaurants.

As mentioned above, the core essence of the TAM is to measure the likelihood of using a technology system (rather than how the system is actually used), which is consistent with the goal of this study (the intention to use restaurant subscription platforms). As surplus food has potential impacts on environmental sustainability, this study intended to probe consumers’ technological acceptance of environmental sustainability issues, such as restaurant subscription platforms, which have not yet been implemented in Taiwan, through the TAM. In other words, the researchers explored the key factors influencing consumers’ intention to use the restaurant subscription platform by adding two relevant variables, namely, the new environmental paradigm and environmental behavior, apart from the three main TAM constructs of perceived usefulness, perceived ease of use, and attitude toward using, to predict consumers’ acceptance of the restaurant subscription platform. Specifically, the researchers planned to investigate the intention of consumers eating out in using restaurant subscription platforms by taking the consumers eating out in Taiwan as the study subjects. The results of this study can clarify the causal relationship between the perceptions and behaviors of consumers eating out towards restaurant subscription platforms and facilitate suggestions to reduce food waste. It is expected that the proposed suggestion can reduce the environmental impact of surplus food [3] and improve economic efficiency [4].

## 2. Literature Review

### 2.1. Sharing Economy and Restaurant Subscription Services

Past findings revealed that when expectations for the quantity and quality of a restaurant’s products are growing, it drives restaurant operators to prioritize consumer satisfaction over food waste [25]. Apart from that, fierce market competition can force restaurant operators to diversify their menu offerings, often at the expense of wasted food [1]. Therefore, food waste should be considered as avoidable. The sharing economy is a business model in which everyone participates by establishing a sharing platform due to oversupply. To solve the problem of food waste caused by food consumption, a sharing platform can be established to reduce food loss and waste.

The subscription service refers to the subscription request sent by the subscriber to the system providing services (Publisher). The system will transmit the service to the subscriber according to the subscription request. The Publish Subscription will be used to reduce the information users need to find [26]. Additionally, Baxter [27] defined a subscription service as an agreement between consumers and enterprises on repeated purchases of products or services. Jamie Lin, the founder of AppWorks, pointed out that subscription is simply a concept combining mass merchandising and long-term contracts [28]. Due to the widespread use of network technologies, regular delivery of newspapers and magazines and home delivery of various kinds of food or supplies are gradually integrated with the subscription business model. In this regard, this model already operates in our daily life [29].

The rising subscription service model is mutually beneficial to the supply and the demand ends, and it also makes a great contribution to environmental protection [30]. To stimulate consumers to buy new products based on the designed service life of products, most of them, including electronic products, are designed to be easily damaged once the warranty period expires, bringing a considerable amount of electronic waste annually across the globe. Nevertheless, if a subscription model is adopted, such a model may be completely changed when all maintenance costs fall on the supplier [31]. The table and chair unsubscription method launched by IKEA, a Swedish furniture brand, has started the trend of long-term and short-term subscription of services, causing many world-renowned enterprises to follow this model. Furthermore, fast fashion in recent years is also an example, for which new clothing is launched every week or every season, but later, some people began to think about where the recycled new clothing should be placed. After protests by environmental protection groups, the new clothes off the shelf finally have a new place to go, and hence, a subscription service platform for environmental protection emerged [32].

It can be seen from the above that the average consumption costs for consumers are low, and it is highly convenient for them in terms of subscription platforms. With the influx of many merchants, there are a variety of meals provided on the platform; restaurant operators can also increase exposure, cultivate long-term relationships with customers, and reduce food waste, in addition to generating regular income through the platform. As a result, it can be said to be mutually beneficial to both the supply and demand sides [33,34].

### 2.2. Technology Acceptance Model (TAM)

The Technology Acceptance Model (TAM) developed by Davis [6] is one of the most influential extended models of the Theory of Reasoned Action (TRA). By combining the TAM with the Theory of Planned Behavior (TPB), one can explore the development model of users and their use of information technology and further explain and predict the influencing factors of the acceptance of users in using a specific information technology. It is proposed that perceived usefulness and perceived ease of use are the two basic factors determining the attitude toward using and intention to use regarding information technology. The main variables are explained as follows.

#### 2.2.1. Perceived Usefulness (PU)

Perceived usefulness refers to the extent to which users believe that the use of a specific information technology will improve performance [11]. Users expect that work performance can be improved in the organizational environment and with a particular system applied [35]. Users hold the view that the use of this technology helps improve work performance and benefits their future. When users perceive that the system is helpful, they are motivated to perform more work with the same efforts to improve their performance [36]. Scholars suggested that perceived usefulness means when users perceive greater benefits, such as being faster, they show more positive attitudes toward the use of the system [18].

#### 2.2.2. Perceived Ease of Use (PEU)

Perceived ease of use refers to users’ perceived ease of use for a specific information technology [11]. It means a user’s personal perception of how easy it is to use the system or how effortless it is to adopt the system [37]. Technology systems allow users to perform tasks faster by improving productivity, performance, and efficiency [19]. When users perceive a system to be easier to use, they will have more positive attitudes toward its use [38]. Users perceive the ease of use of technology. As a technology becomes easier to use, users will have more confidence in their self-performance and more positive attitudes toward their systems [36].

#### 2.2.3. Attitude toward Using (ATU)

Davis [11] identified attitude toward using as users’ positive or negative perceptions of using a technology system. Although it is originally used to explain and predict the use of technology in the workplace, it can also be employed to predict consumer behavior in e-commerce, where perceived usefulness, perceived ease of use, and attitude toward use were found to be positively correlated [39]. In addition to different perceptions, users’ behavioral intentions are also affected by their preference for the system in the TAM [40]. Perceived usefulness and perceived ease of use would lead to positive attitudes toward the system, which may enhance the intention to use [41].

#### 2.2.4. Behavior Intention to Use (BIU)

Behavior intention to use refers to users’ intention to use a technology system. When their intention tends to be strong, their intention to use a technology system will be higher and the intention to use will also be influenced by their attitude toward using [11]. The likelihood that an individual, affected by subjective awareness, will adopt a new technology system reflects the strength of the users’ desire to have a behavior and represents their intention to use [42]. If they are willing to devote time and effort and hold a strong intention to use, it means that they are willing to make more effort and take more actions [43].

### 2.3. New Environmental Paradigm (NEP)

The new environmental paradigm (NED) was proposed by Dunlap and Van Liere [44], who suggested that humans are only a member of the natural world, where various resources are limited, and the Earth has its limits, and that humans should recognize the importance of a balanced ecosystem and sustainable development. This paradigm emphasizes sustainable development, peaceful coexistence, and a limited supply of all resources [21,45]. It is an ideology that emphasizes intuitive analysis and personal experience and promotes a mindset that embraces nature, people, and the planet [46].

According to the paradigm, researchers are concerned with the sustainable maintenance of the natural environment and the balanced maintenance of the ecosystem, especially the interaction between environmental sustainability and human existence. The concern for the balance between nature and humans has led to the development of methodologies and research results related to the measurement of the environment, including the reality of limits to growth, anti-anthropocentrism, the fragility of nature balance, the rejection of exemptionalism, and the possibility of an ecocrisis. These problems all involve the interaction between environmental sustainability and human survival [21,22,23,45]. In other words, the focus of relevant research is on whether people can view the relationship between human beings and nature in a more far-sighted and objective way, as well as the sustainability of ecology, instead of treating them in a selfish and profit-oriented manner. For that matter, in-depth exploration of human behaviors and attitudes toward the environment has become a factor and topic that researchers have paid more attention to in recent years.

### 2.4. Environmental Behavior (EB)

In the past, studies related to environmental behavior were more concerned with causal inferences between variables. For example, the predictors of ecological waste reduction, reuse, and recycling differed significantly. As for recycling behavior, it was described as highly normative [20]. Moreover, personal values had a direct impact on behavioral intention and an indirect impact on attitude [47]. In similar studies, personal norms or factors closely related to personal norms (including self-identity and personal values) were found to be correlated with the consumption of sustainable products, such as the purchase of suboptimal (visually imperfect but environmentally friendly) products [48].

In recent years, studies related to environmental behavior have tended to focus on discussions over consumer attitudes, including the impact of consumers’ environmental beliefs on green consumption behaviors and environmental attitudes in general [49], the improvement of household-related food waste prevention behaviors [50], consumers’ environmental attitudes and pro-environmental behaviors [51], environmental conservation attitude and attitude toward nature [52], green product attitudes and green product purchase intention [53], the influence of environmental beliefs and social factors on green purchase attitudes [54], and the impact of the formulation and implementation of environmental policies on enhancing environmental behaviors [55]. From this trend, it can be seen that with the growing explorations of behaviors and attitudes, researchers have attached importance to measuring the values of individuals on environment-related issues, that is, environmental attitudes and related behaviors.

### 2.5. Relationships between Variables

#### 2.5.1. Perceived Usefulness and Attitude toward Using

Perceived usefulness means that when users perceive greater benefits, such as being faster, they will show more positive attitudes toward using the service [13]. It has been suggested that perceived usefulness affects the attitude of using mobile commerce and the intention to use it when travelers adopt it [56]. Chang and Chiang [57] investigated the relationship between customer value, perceived risk, and intention to use, and discovered that the usefulness of mobile payment services has a significant positive effect on the attitude toward using, and the attitude to use mobile payment services also had a significant positive effect on the intention to use. Therefore, enhancing the perceived usefulness of mobile payment can affect customers’ attitude toward using mobile payment, thus improving the intention to use. For the behavioral intention of airport passengers to use self-check-in services, perceived usefulness also had a positive effect on attitude and behavioral intention [58]. In a study by Hsieh [36] on the use of responsive websites by older people, it was verified that perceived usefulness and attitude toward using were positively correlated. All of the above studies indicated that the perceived usefulness of a technology product or technology positively affects consumers’ attitudes toward use. Consequently, the following hypothesis was proposed in this study:

**H1.**  *The perceived usefulness of restaurant subscription platforms has a significant positive effect on consumers’ attitude toward using.*

#### 2.5.2. Perceived Ease of Use and Attitude toward Using

Technology systems allow users to perform tasks faster by improving productivity, performance, and work efficiency [19]. The more user-friendly a system is, the more positive users’ attitudes toward its use are [38]. Wang and Hsu [59] found that perceived ease of use is the most important factor influencing community leaders’ actual use of the Taiwan Community Link, while attitude toward using is affected by perceived usefulness and perceived ease of use of the website. The perceived usefulness and perceived ease of use of Community Access were the most important factors influencing community leaders’ actual use of the Taiwan Community Access website. The perceived ease of use of mobile payment is positively correlated with the attitude toward using, and the latter is positively correlated with intention to use [60]. Additionally, in Lin’s [43] exploration of consumers’ intention to use delivery apps, he discovered that the more consumers agree with the perceived ease of use, the more likely they are to agree that delivery apps are in line with their lifestyles and then have a sense of superiority and even recommend others to use them. It can be understood from the above findings that the perceived ease of use of a technology product or a technology positively affects consumers’ attitudes toward use of it. Therefore, the following hypothesis was proposed in this study:

**H2.**  *The perceived ease of use of restaurant subscription platforms has a significant positive effect on consumers’ attitude toward using.*

#### 2.5.3. New Environmental Paradigm and Attitude towards Use

A scholar in Taiwan suggested that the more supportive one is for a new environmental paradigm, the more it affects one’s attitude toward the environment and the higher one’s self-assessed likelihood of adopting environmental behavior [61]. Similarly, a study in Japan [62] investigated whether there is a correlation between Japanese people’s awareness of the environment, attitudes toward conservation, and environmental behavior intentions and explored the concept of their environmental awareness in terms of respect for resources. This study concluded that an effective way to promote environmental protection is to make people be more concerned about the environment and to have a positive attitude toward conservation. Past studies have shown that new environmental paradigms have a significant positive effect on the purchase attitudes toward green products [63]. Additionally, environmental responsibility norms, environmental beliefs, and perceived usefulness directly influence individuals’ attitudes and intentions to use green information technologies, such as environmentally friendly cars and energy-efficient home appliances or systems [64]. As a result, the following hypothesis was derived in this study:

**H3.**  *A better new environmental paradigm for consumers has a significant positive effect on their attitudes toward use of the restaurant subscription platform.*

#### 2.5.4. Environmental Behavior and Attitude toward Using

The Earth’s environment is being increasingly damaged. In order to improve the living environment, it is necessary to have environmentally friendly behaviors or activities, such as recycling, energy conservation, and environmental protection [65]. In October 2018, the Intergovernmental Panel on Climate Change (IPCC) published that excessive consumption results in habitat loss, biodiversity loss, pollution, and climate change and therefore consumer behavior and attitude need to be changed to advance sustainable environmental and economic growth [66]. Wang [67] reflected that in order to change people’s behaviors towards environmental pollution, we can provide specific solutions to increase people’s confidence and have a correct attitude so as to avoid actions polluting the environment. Chiu [68] indicated that among the factors influencing environmentally friendly behaviors, the more people have a positive ecological view, the better their behavioral intentions and behavioral performance, the better their environmental awareness and attitudes, and the higher the price they are willing to pay for green products. Accordingly, based on the above findings, the following hypothesis was proposed in this study:

**H4.**  *Consumers’ better environmental behavior has a significant positive effect on their attitude toward using restaurant subscription platforms.*

#### 2.5.5. Attitude toward Using and Intention to Use

In addition to different perceptions, users’ behavioral intentions are also affected by their preference for the system in the TCM [40]. Perceived usefulness and perceived ease of use would lead to positive attitudes toward the system, which may enhance the intention to use [41]. It has been suggested that consumers’ attitudes toward using smart wearable devices for mobile payment have a significant positive relationship with behavioral intentions [69]. In a study where travelers adopted the behavior model of mobile commerce, perceived usefulness and perceived ease of use positively influenced attitude toward using, and attitude toward using also positively influenced behavioral intention [56]. Additionally, attitude towards shopping was also significantly related to purchase intentions [69]. Furthermore, consumers’ attitudes toward use of mobile banking also affect intention to use [17]. These findings indicated when a technology product or technology is used, a positive attitude toward using the product will enhance the intention to use it. Thus, consumers’ attitudes toward use will positively influence their intention to use. In consequence, the following hypothesis was proposed in this study:

**H5.**  *Consumers’ attitude toward using restaurant subscription platforms has a significant positive effect on their intention to use.*

#### 2.5.6. Perceived Usefulness, Attitude toward Using, and Intention to Use

Users’ perceived usefulness of a technology product or technology (e.g., it enhances performance and is helpful in life) affects their attitude toward using (e.g., they may consider it as a wise choice and the right way to do things). Accordingly, when the usefulness increases, users will have a more positive attitude toward using the QR-CODE, thus affecting their intention to use it. In the study of consumers’ behavioral intention to use QR-CODEs for online shopping, it was found that users’ attitude toward using has a mediated effect on the relationship between perceived usefulness and behavioral intention [70]. Moreover, in an exploration of users’ intention to use smart speakers, it was found that their attitude toward using has a partial mediated effect on perceived usefulness and behavioral intention to use [71]. In Wu’s [72] study of the key factors influencing mobile payment, attitude toward using also plays a mediating role between perceived usefulness and intention to use. According to the above literature, attitude toward using a technology product or technology has a mediated effect on perceived usefulness and intention to use. Consequently, the following hypothesis was proposed in this study:

**H6.**  *Consumers’ perceived usefulness of restaurant subscription platforms affects their intention to use through their attitude toward using.*

#### 2.5.7. Perceived Ease of Use, Attitude toward Using, and Intention to Use

When users find it easy to use a technology product or technology (easy and effortless to operate), their attitude toward using will be affected (e.g., they perceive it as a positive and good feeling). When it is easier to use, users will have a more positive attitude toward using, thus affecting their intention to use. In a study by Liao [73] on the factors influencing the loyalty of using mobile Internet banking, it was pointed out that the attitude toward using mediates brand equity, perceived usefulness, and perceived ease of use and thus affects loyalty. Kuo [74] explored the intention to use third-party payments through TAM, showing that when consumers perceive that third-party payment platforms have easy-to-understand online shopping processes, easy-to-learn interfaces, and easy-to-operate system functions, they will also have a positive attitude toward such platforms, having a high intention to use them. In addition, the attitude toward using mobile payment plays an important mediation role in perceived ease of use and intention to use, and it is also an important factor influencing intention to use [60]. The above studies indicated that attitude towards use of a technology product has a mediated effect on perceived ease of use and intention to use. Therefore, the following hypothesis was proposed in this study:

**H7.**  *Consumers’ perceived ease of use of restaurant subscription platforms has an impact on their intention to use through their attitude toward using.*

#### 2.5.8. New Environmental Paradigm, Attitude toward Using, and Intention to Use

The findings of Liao et al.’s [75] study show that consumers’ general attitudes, attitudes toward money and the environment, subjective norms, personal norms, and perceived behavioral control positively influence consumers’ intention to reduce food waste. Apart from that, a study on the relationship between teachers’ attitudes toward sustainability, air pollution risk arguments, and air pollution prevention behaviors pointed out the more positive teachers’ attitudes toward environmental sustainability, the more likely they are to agree with arguments related to air pollution risks and the more likely they are to engage in air pollution prevention, and thus it is necessary to make teachers emphasize their attitude toward sustainability [76]. Based on the above findings, this study included the new environmental paradigm into the TAM to observe the relationship between users’ new environmental paradigm (environmental attitude) in the attitude toward using and intention to use restaurant subscription platforms and suggested the following hypothesis:

**H8.**  *The new environmental paradigm influences their intention to use, through their attitude toward using, when consumers use restaurant subscription platforms.*

#### 2.5.9. Environmental Behavior, Attitude toward Using, and Intention to Use

If consumers have a more positive attitude toward using a technology product or technology, they will have more positive evaluations, better environmental behavior, and higher intention to use the technology product or technology. Liang [77] pointed out that consumers’ positive brand attitude toward processed surplus food products would enhance their intention to purchase. Furthermore, the mediated effect of brand attitude in the model was also verified, as altruistic consumers would be more receptive to processed surplus food products and enhance their purchase intention. Therefore, the following hypothesis was inferred in this study:

**H9.**  *The environmental behavior of consumers using restaurant subscription platforms affects their intention to use through their attitude toward using.*

## 3. Methodology

### 3.1. Ethical Statement

This study was conducted based on the guidelines of the Declaration of Helsinki. Subjects were informed of the purpose and nature of the research and provided their informed consent. The approval to conduct this study was obtained from the Ethics Committee of Chung Shan Medical University, Taiwan (IRB Number: CS1-24163, Date: 1 August 2020).

### 3.2. Sample and Data Collection

The subjects of this study are employees who often eat out. A formal questionnaire was conducted in paper form. The questionnaires were sent out in a snowballing manner with the help of colleagues and friends who know people who habitually eat out. Additionally, a survey targeting the group who dine out was conducted at a restaurant, with the purpose of improving the recovery rate of valid questionnaires in a short period of time. The formal questionnaire was carried out in mid-August 2020 for a period of two months. A total of 420 questionnaires were distributed and 400 questionnaires were collected and 369 valid ones were returned. After deducting the invalid ones, the number of valid questionnaires was 369, with a valid response rate of 92.25%. In the descriptive analysis of the sample, most of the respondents were females (*N* = 192, 52.0%; males: *N* = 177, 48.0%); in age distribution, those aged 40–49 were the majority (*N* = 118, 32.0%), followed by 30–39 (*N* = 91, 24.7%); in terms of whether to pay attention to environmental protection issues, there were people who paid attention to it (*N* = 310, 84.0%), but there were those who did not (*N* = 59, 16.0%). Sample characteristics are presented in Table 1.

### 3.3. Statistical Analysis

Statistical package for social science (SPSS) (IBM Corp.: New York, NY, USA) and Analysis of Moment Structure (AMOS) version 22 (IBM Corp.: New York, NY, USA) were used for data analysis in a seven-step process. Descriptive statistics summarized the data, followed by reliability analysis with Cronbach’s alpha to ensure internal consistency. Confirmatory Factor Analysis (CFA) validated the measurement model, and Pearson correlation assessed variable relationships. Structural Equation Modeling (SEM) tested the hypothesized relationships and model fit using various indices. For mediation analysis, we followed the Preacher and Hayes method to assess whether the mediator reduced the direct effect of the independent variable. Bootstrapping with 1000 resamples confirmed the mediation effect’s significance with a 95% confidence interval.

### 3.4. Measurement

**Figure 1:**
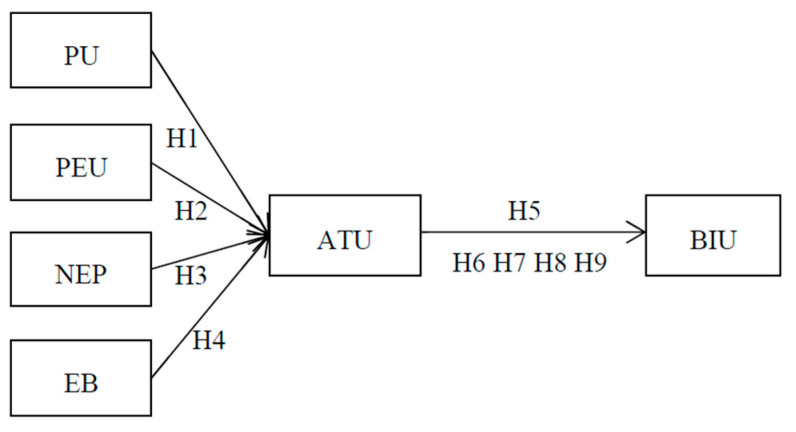
Research framework.

This study investigated the relationship between perceived usefulness, perceived ease of use, new environmental paradigm, environmental behavior, attitude toward using, and intention to use, with the TAM proposed by Davis [11] as the theoretical basis. Figure 1 presents the research framework for exploring the relationship between perceived usefulness, perceived ease of use, attitude toward using, intention to use, new environmental paradigm, and environmental behavior of restaurant subscription platforms. The three-item scale in the perceived usefulness section of the questionnaire included items such as “I feel that using the restaurant subscription system allows me to quickly receive information about the restaurant (restaurant location, distance, and menu)”, and was adapted from Davis with a Cronbach’s α of 0.772 [11]. The three-item scale in the perceived ease of use section of this survey included items such as “I think it is easy to use a restaurant subscription app”, and was adapted from Davis with a Cronbach’s α of 0.877 [6]. The three-item scale in the attitude toward using section of this survey included items such as “I’m interested in restaurant subscription as opposed to the traditional way of spending money in a restaurant”, and was adapted from Davis with a Cronbach’s α of 0.857 [11]. The four-item scale in the intention to use section included items such as “If a restaurant subscription-related platform is launched, I would want to use it”, and was adapted from Davis with a Cronbach’s α of 0.925 [11]. The three-item scale in the new environmental paradigm section of the questionnaire included items such as “I think the space and resources on the earth are limited, so we should not waste food”, and was adapted from Dunlap and Van with a Cronbach’s α of 0.880 [44]. The two-item scale in the environmental behavior section of this survey included items such as “If the restaurant subscription system provides surplus food, I might want to have a try”, and was adapted from Hines with a Cronbach’s α of 0.940 [78]. The Cronbach’s α coefficients should be at least 0.50 and preferably greater than 0.70 [79], and on each scale, the questionnaire is measured on a five-point Likert scale ranging from 1 (strongly disagree) to 5 (strongly agree). Details about the source of the questionnaire items are highlighted in Table 2.

## 4. Results

### 4.1. Reliability and Validity

The main function of reliability analysis is to examine whether the measurement results of variables are stable and consistent and how stable and consistent they are. If the value of the reliability coefficient is high, the intrinsic reliability of the measurement is consistent and reliable [80]. The Cronbach’s alpha value should be at least greater than 0.50 and preferably greater than 0.70 [81]. In this study, the Cronbach’s α in all constructs is greater than 0.7 (see Table 3 for details), indicating that the measurement tool was reliable. In other words, all measurement items in the six constructs were internally consistent and highly stable for the questionnaire. The higher the composite reliability (CR), the higher the proportion of true variance to the total variance. That means there would be higher internal consistency, which could be regarded as the internal consistency of the construct, and CR should be higher than 0.6 [82]. The CR of the variables in this study ranged from 0.78 to 0.94, indicating that this model had good internal consistency. The average variance extracted (AVE) refers to the degree to which all the measured variance in the potential variables can explain the potential variables, which means the higher the AVE, the higher the degree to which the potential variables are explained by the variance in the measured variables. The AVE for each factor was between 0.55 and 0.89, which is higher than the recommended benchmark of 0.5 [82]. The results show that the CRs of the constructs are greater than 0.6, reflecting that each construct was consistent and had high reliability. The AVE values are also greater than 0.5, indicating good convergent validity. Means, standard deviations, and correlations among the constructs are presented in Table 4. Perceived usefulness and perceived ease of use have a significant positive correlation with attitude toward using (γ = 0.663, γ = 0.670, *p* < 0.01). That means when consumers believed there was higher perceived usefulness or perceived ease of use of the restaurant subscription platform, their attitude toward using the platform tended to be positive. There was also a significant positive correlation between attitude toward using and intention to use (γ = 0.790, *p* < 0.01), which means when consumers had a more positive attitude toward the restaurant subscription platform, their intention to use it would be stronger. Both new environmental paradigm and environmental behaviors had a significant positive correlation with attitude toward using (γ = 0.265, γ = 0.276, *p* < 0.01), which means consumers’ attitude toward using would be more positive if they had better new environmental paradigms and environmental behaviors.

### 4.2. Structural Equation Modeling and Empirical Analysis

**Figure 2:**
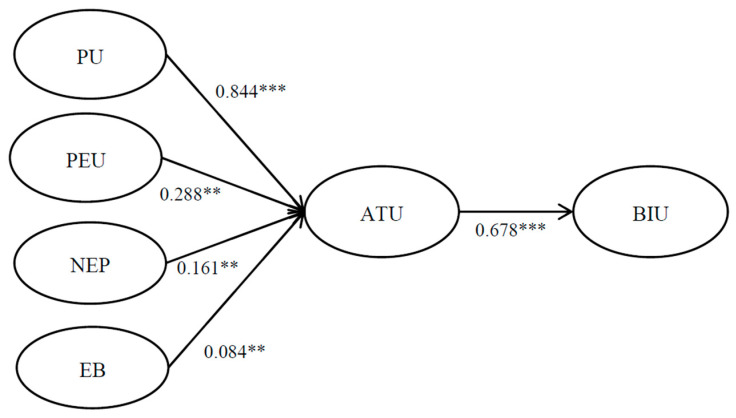
Results of structural equation modeling Note: ** *p* < 0.01; *** *p* < 0.001.

This study applied SEM using AMOS 22.0 to assess the path relationships among green perceived value, animal welfare value, attitude, purchase intention, and product knowledge. The results indicate that the measurement model provided a good fit for the data (χ^2^/df = 1.996, GFI = 0.934, AGFI = 0.906, NFI = 0.953, RFI = 0.940, IFI = 0.976, CFI = 0.976, SRMR = 0.032, RMSEA = 0.052). The χ^2^/df ratio is below the value of 3 [83]; the GFI, AGFI, NFI, RFI, IFI, and CFI exceeded the recommended threshold of 0.90 [84,85]; the SRMR value was less than 0.05, indicating a good fit [86]; and the RMSEA value was between 0.05 and 0.08, reflecting a moderate fit [87]. This indicated that the approach used in this study for modeling the examined data was appropriate. The hypothesis testing results from the model data are provided in Figure 2 and Table 5.

The path coefficients between perceived usefulness, perceived ease of use, new environmental paradigm, environmental behavior, and attitude toward use all reached significant levels (γ = 0.844, *p* < 0.001; γ = 0.288, *p* < 0.01; γ = 0.161, *p* < 0.01; γ = 0.084, *p* < 0.01), as did the path coefficient between attitude toward use and intention to use (γ = 0.678, *p* < 0.001). This indicates a significant positive relationship between perceived usefulness, perceived ease of use, new environmental paradigm, environmental behavior, and attitude toward use, as well as between attitude toward use and intention to use within the context of restaurant subscription platforms. Specifically, higher levels of perceived usefulness, perceived ease of use, new environmental paradigm, and environmental behavior are associated with a more favorable attitude toward using the platform, which in turn leads to a stronger intention to use it. Moreover, the R-squared value for “environmental behavior”, “perceived ease of use”, “new environmental paradigm”, and “perceived usefulness” in predicting “attitude toward use” is 0.564, indicating that these variables collectively explain approximately 56.4% of the variance in attitude toward use. In comparison, the R-squared value for “attitude toward use” in predicting “intention to use” is 0.624, suggesting that attitude toward use accounts for approximately 62.4% of the variance in intention to use. Thus, the empirical data supported hypotheses H1–H5. This analysis underscores the strong predictive power of these factors in shaping both users’ attitudes toward and intentions to use restaurant subscription platforms.

### 4.3. Mediated Effect Analysis

Mediated effect refers to the path effect of the independent variable X indirectly affecting the dependent Y after affecting the mediator variable M [88]. Bootstrapping was applied in this study to obtain the confidence interval of indirect effect and to simultaneously test whether the hypothesis of mediated effect is true. Bootstrapping is used to treat the sample itself as a population to keep on sampling repeatedly (e.g., 1000 times). These 1000 estimates will be in a new distribution, and a confidence interval can be derived. It is then seen whether this confidence interval passes through 0. If it does not pass through 0 and the *p*-value is less than 0.05, it is more certain to be statistically significant [89]. In PU → ATU → BIU, the indirect effect was significant, the direct effect was significant in PU → BIU, and the total effect was also significant in PU → BIU. Consequently, attitude toward using did play a partially significant mediation role in the relationship between perceived usefulness and intention to use. There was no indirect effect in PEU → ATU → BIU, no direct effect in PU → BIU, and no significant total effect in PEU → BIU. Accordingly, attitude toward using had no mediated effect on the relationship between perceived ease of use and intention to use. The indirect effect was significant in NEP → ATU → BIU, and there was no direct effect in NEP → BIU. Consequently, attitude toward using played a fully mediating role in the relationship between new environmental paradigms and intention to use. The indirect effect was significant in NEP → ATU → BIU, while there was no direct effect in NEP → BIU. Therefore, attitude toward using did play a full mediation role in the relationship between environmental behavior and intention to use. Thus, H6, H8, and H9 were supported by empirical data (see Table 6 and Table 7 for details).

## 5. Conclusions and Suggestions

### 5.1. Conclusions

#### 5.1.1. The Relationship between Consumers’ Perceived Usefulness and Their Attitude toward Using Restaurant Subscription Platforms

Consumers dining out feel that the higher the perceived usefulness of the restaurant subscription platform is, the better their attitude toward using the platform will be. In other words, when these consumers reckon that such platforms can help meet their needs in life (eating out) or improve efficiency (convenient for dining), it will be easier for them to accept the platforms. That fully conforms to the research results of the TAM proposed by Davis [11] and is consistent with the findings of Lin and Liu [56] and Chang and Chiang [57]. That is, when users find it more beneficial in using a technology system, they will have a positive feeling about the technology product and have a more positive attitude toward using it.

#### 5.1.2. The Relationship between Consumers’ Attitude toward Using and Their Perceived Ease of Use of Restaurant Subscription Platforms

Consumers eating out feel that the higher the perceived ease of use of the restaurant subscription platform is, the better their attitude toward using the platform will be. In other words, when they find it easier to use (simple operation) the platform, it will be more acceptable to them. That is fully consistent with the research results of the TAM proposed by Davis [11] and in line with the findings of Wang and Hsu [59], Lin [43], and Chang and Lo [60]. That is to say, the higher the perceived ease of use of a technology system is, the more positive an individual’s attitude toward the new technology will be.

#### 5.1.3. The Relationship between Consumers’ New Environmental Paradigm and Their Attitude toward Using Restaurant Subscription Platforms

The new environmental paradigm can affect the attitude toward using restaurant subscription platforms; when consumers dining out have a good new environmental paradigm, their attitude toward using the platform tends to be more positive. In other words, when these consumers have a more objective awareness of the environment or attach more importance to the sustainable development of resources, it will be easier for them to accept the platform. That is, when users have a new environmental paradigm, their attitude toward new technology will be more positive, which is consistent with the findings of Yeh [63] and Yoon [64].

#### 5.1.4. The Relationship between Consumers’ Environmental Behavior and Their Attitude toward Using Restaurant Subscription Platforms

Environmental behavior can also affect the attitude toward using the restaurant subscription platform; when consumers dining out have good environmental behaviors, their attitude toward using the platform tends to be more positive. In other words, if these consumers are willing to take actions to tackle and prevent environmental problems, including paying or exchanging for surplus food to avoid food waste, it will be easier for them to accept such a platform. That is to say, when users have better environmental behaviors, their attitude toward new technology will be more positive, which is consistent with the findings of Wang [67] and Chiu [68].

#### 5.1.5. The Relationship between Consumers’ Attitude toward Using and Their Intention to Use Restaurant Subscription Platforms

When consumers eating out have a better attitude toward the restaurant subscription platform, they will have a stronger intention to use the platform. In other words, the more positive their attitude toward the platform is, the stronger their intention to use it in the future will be. That is fully consistent with the research results of the TAM proposed by Davis [11] and in line with the research findings of Pappa et al. [90], Putri et al. [17], and Yu et al. [69] That is to say, the more positive users’ attitude toward using a technology system, the stronger their intention to use that new technology.

#### 5.1.6. The Mediated Effect of Consumers’ Attitude toward Using Restaurant Subscription Platforms between Their Perceived Usefulness and Intention to Use

The attitude of consumers has a partially mediated effect on the relationship between perceived usefulness and intention to use. The research results show that if consumers eating out have a more positive attitude toward the restaurant subscription platform, it will be easier for them to accept it, they will feel greater benefits from the platform, and they will have a stronger intention to use it. This view is consistent with the findings of Wu [72], Huang [70], and Cheng [71].

#### 5.1.7. The Mediated Effect of Consumers’ Attitude toward Using Restaurant Subscription Platforms between Their Perceived Ease of Use and Intention to Use

Consumers’ attitude has no mediated effect between their perceived ease of use and intention to use The research results indicate that the acceptance and evaluation of the restaurant subscription platform by those eating out will not affect their ease of use of the platform, nor will they affect their intention to use it This opinion does not conform with the research findings of Kuo [74] and Liao [73], but it is consistent with the findings of Chang [91], who posed that the attitude toward using water-based runway products has no mediated effect in the relationship between the perceived ease of use and intention to use, and Cheng [92], who argued that attitude toward using digital financial platforms has no mediated effect on the perceived ease of use and intention to use.

#### 5.1.8. The Mediated Effect of Consumers’ Attitude toward Using Restaurant Subscription Platforms between Their New Environmental Paradigm and Intention to Use

The attitude of consumers has a fully mediated effect between the new environmental paradigm and intention to use. The research results show that the new environmental paradigm (attitude toward the environment) of consumers eating out and their intention to use can be fully affected by their attitude toward using the platform. This is consistent with the research findings of Shein et al. [76].

#### 5.1.9. The Mediated Effect of Consumers’ Attitude toward Using Restaurant Subscription Platforms between Their Environmental Behaviors and Intention to Use

The attitude of consumers has a fully mediated effect between their environmental behaviors and intention to use. The research results reflect that if consumers dining out have a more positive attitude toward using and positive evaluation of the restaurant subscription platform, they will have better environmental behavior. That is to say, they will be willing to purchase surplus food or ingredients, showing a stronger intention to use the platform. This view is consistent with the findings of Liang [77].

### 5.2. Suggestions

#### 5.2.1. Develop a Restaurant Subscription Platform with Sufficient and Correct Information

When consumers find it convenient and useful to dine out using the restaurant subscription platform, they will have a more positive attitude toward using the platform. Therefore, it is suggested that restaurant operators should provide sufficient relevant information [93] and pay attention to the correctness of the content [94] when developing and using restaurant subscription platforms to improve the usefulness of the apps.

#### 5.2.2. Design a Restaurant Subscription Platform with an Interface That Is Easy to Operate

When consumers feel that the interface of the restaurant subscription platform is simple and easy to operate, they will have a more positive attitude toward using it. Accordingly, it is suggested that when developing and using restaurant subscription system platforms, restaurant operators should design the control interface of the app to be easy to use [95] and make it conform to the habits of the public in using it so that they can operate it and subscribe to it smoothly.

#### 5.2.3. Support the Concept of Sustainability and Maintain a Friendly Environmental Attitude

When consumers feel that the restaurant subscription platform is conducive to environmental sustainability and can reduce food waste, they will have a more positive attitude toward using it. Consequently, it is suggested that restaurant operators should support the concept of sustainability or maintain a friendly environmental attitude and behavior [96] to enhance consumers’ positive attitude toward using the platform.

#### 5.2.4. Add Customized Surplus Food without Safety Concerns for Subscription

When consumers feel the restaurant subscription platform can reuse surplus food, redistribute the ingredients, and solve or prevent environmental problems, they will have a more positive attitude toward using. Therefore, it is suggested that restaurant operators can add customized surplus food without safety concerns and the option of subscribing to the platform for surplus ingredients [97], which enables consumers to have a more positive attitude toward using the platform.

#### 5.2.5. Make Good Use of Social Media to Promote the Restaurant Subscription System and Platform

This study found that the attitude toward using the restaurant subscription platform was the key to the intention to use the platform. When the favorability of consumers eating out toward use of restaurant subscription platforms increased by 1, their intention to use it would increase to 0.79, which illustrates that attitude was the highest variable affecting the intention to use it. As a result, making consumers’ attitudes toward using more positive means to enhance their sense of identity with the platform, thus making their intention to use stronger. In the past two to three years, the restaurant subscription system has become popular in the Japanese food industry and created a trend. Since this business consumption model is not yet prevalent in Taiwan, restaurant operators can make use of social media to advertise [98], promote their restaurant subscription platforms, and increase the exposure rate [99] to raise consumers’ awareness and interest in the platform. They can also launch marketing campaigns and promotional seminars for restaurant subscription platforms to draw more attention [100] and elicit positive attitudes toward using from the public regarding this new restaurant service so that they will be more willing to continue in the subscription.

#### 5.2.6. Continuously Better Customer Experience of Using the Restaurant Subscription Platform

The original intention to use the restaurant subscription platform caused by the convenience of use can be strengthened or changed by making consumers’ attitude toward using the platform more positive. Consequently, restaurant operators should strive to better customers’ experience of using such a platform [101], increase their usage, and enhance their sense of identity to build customer loyalty. In this way, a positive attitude toward the platform will be generated, and consumers will find it convenient and useful, thus enhancing their intention to use.

#### 5.2.7. Add Innovative Elements to the Application Interface to Motivate Users

The original intention to use the restaurant subscription platform arising from simple operations cannot be changed by making consumers’ attitude toward using more positive. This may be due to the fact that mobile applications (apps) are familiar to most consumers and are less challenging to operate [102]. Therefore, it is suggested that operators can add some innovative design elements to the platform [95] to motivate consumers to use it, thus enhancing their intention to use it.

#### 5.2.8. Introduce the Concept of Restaurant Sustainability to Groups with Strong Environmental Awareness

Making consumers’ attitude toward using the restaurant subscription platform positive can strengthen or change the original intention to use it driven by the new environmental paradigm. In consequence, the operators can change their attitude toward the restaurant subscription platform by introducing the concept of sustainability and emphasizing the functions of the platform to groups with strong environmental awareness through the community [103]. The intention to use the platform will also be stronger.

#### 5.2.9. Cooperate with Celebrity Chefs to Promote or Participate in the Sustainable Spiritual Food Program

Making consumers’ attitude toward restaurant subscription platforms more positive can strengthen or change their original intention to use caused by personal environmental behavior. Therefore, new entrepreneurs can invite influential chef celebrities to publicize or participate in programs, like the sustainable spiritual food program [104,105] initiated by Chef Massimo Bottura, to gain more support and network popularity (favorable review influence). They can also develop franchise-based or eco-friendly restaurant subscription platforms, match multiple restaurant operators and consumers, or connect surplus food providers (restaurant operators) with those in need (consumers) [106], enabling users to maintain a positive attitude toward the platform to continue to subscribe to it.

#### 5.2.10. Improve Food Resource Utilization and Sustainability

To provide a comprehensive understanding of the operational model of surplus food restaurants, factors such as platform dependency, potential competition, market saturation, service quality and consistency, food safety issues, and the environmental impact of single-use packaging need to be thoroughly analyzed. Furthermore, to offer practical recommendations for improving food resource utilization and sustainability, governments should also implement policies to reduce plastic usage, highlighting the importance of addressing environmental concerns.

## 6. Contributions and Limitations

### 6.1. Contributions

#### 6.1.1. Academic Contributions

There is a considerable amount of literature on the use of the Taiwanese and international TAMs. Taiwan ranked in the top three (294 papers, 5268 citations) among the countries and regions conducting relevant studies with the TAM from 2010 to 2020; the top three areas for exploring consumers’ intention to use a new technology were e-commerce, internet banking, and social media [9]. However, these documents focus less on subscription services, especially in the field of using subscription services in the catering industry. Therefore, this study applied the TAM to explore the relationship between perceived usefulness, perceived ease of use, attitude toward using, and intention to use restaurant subscription platforms, which can serve as a reference for subsequent studies.

Food waste is a complex and widely discussed issue in all areas of society today. In most cases, food waste occurs at the end of the production chain, namely meal preparation and distribution. To reap the environmental benefits of the sharing economy (where goods are used efficiently), a restaurant subscription platform can be used to prepare and distribute surplus food. This raises questions about what consumers’ intention to use the platform is. Accordingly, this study proposed to extend the TAM by adding two variables, a new environmental paradigm and environmental behavior, to understand consumers’ environmental attitudes and acceptance of surplus food. It can serve as a reference for subsequent studies as this study combines with the sustainability of catering consumption.

#### 6.1.2. Practical Contributions

In the 2030 Sustainable Development Goals published by the United Nations, countries are instructed to encourage consumers to change their consumption habits to ensure food sustainability and to reduce food loss and waste so as to lower the negative impacts on the environment [107]. In this regard, it is necessary for the catering industry and consumers to actively cooperate to reduce food loss and waste. The subscription service is an emerging business model for restaurants and is not yet common in Taiwan; restaurant operators can use the results of this study as a reference for their decision to use subscription services to create another channel for generating recurring revenue. New entrepreneurs can evaluate whether to develop this type of subscription platform and offer a convenient and economical option for consumers eating out. Moreover, the study results can also be used as a basis for governments, enterprises, or related organizations to develop policies or take measures to reduce surplus food, to actively promote education for all, to advocate corporate social responsibility, and to ensure food waste is reduced.

### 6.2. Limitations

Firstly, more than half of the subjects in this study live in central Taiwan and one-third are educators, resulting in a limited sample size which makes the study results unrepresentative of the whole picture of all consumers, which is one of the limitations of this study. It is suggested that future researchers can expand the sample size by including other groups such as young people, who are usually more familiar with new technologies and may have different views on new business models [108] so as to make data analysis more accurate. Additionally, this study was only conducted in Taiwan. Compared to Taiwan, where the subscription service is still in its infancy, Japan has experienced a restaurant subscription trend since 2018. However, cross-cultural research can be carried out in the future to compare consumers’ intentions to subscribe in different countries. Furthermore, this study was conducted with the aim of exploring consumers’ intention to use the service. Subjects can be extended to restaurant operators and new entrepreneurs in the coming years. In addition to the questionnaire survey for data collection, qualitative interviews can also be conducted with the respondents to further probe the factors influencing the intention to use the subscription service provider.

Secondly, as restaurant subscription systems are still in the emerging stage in Taiwan and most consumers remain unfamiliar with this service concept and platform technology, respondents can only fill out the questionnaire based on their own perceptions and imagination. Although textual descriptions were used and Japanese platforms were introduced in this study, the participants’ answers might not be fully representative and correct. Additionally, it was found that some consumers had misunderstandings about the reuse or distribution of surplus food, resulting in limited or incorrect answers. It is expected that future studies will not only provide more detailed descriptions of restaurant subscription services and reuse of surplus food but also include product involvement or product knowledge variables as mediators or moderating variables between attitude toward using and intention to use [109,110] in order to make the study results more meaningful.

Finally, despite the important variables of the TAM and the variables related to environmental sustainability included in this study, some relevant factors have yet to be fully included. For that matter, it was suggested that some additional variables could be added to the TAM, such as the specific variables of trust and security in e-commerce [111,112,113,114], apart from perceived usefulness and perceived ease of use. Accordingly, the above variables may be explored in future studies to gain a more comprehensive understanding of how consumers accept new technologies.

## Figures and Tables

**Table 1:** Sample characteristics.

*N* = 369	Item	*N*	Percentage
Gender	Male	177	48.0%
	Female	192	52.0%
Age	Under 19	15	4.1%
	20–29	56	15.2%
	30–39	91	24.7%
	40–49	118	32.0%
	50–59	82	18.4%
	Above 60	21	5.7%
Paid attention to environmental issues	Yes	310	84.0%
	No	59	16.0%

**Table 2:** Constructs/variables and corresponding measuring statements included in the questionnaire.

Construct/Variable	Number of Statements	Measuring Items	Sources of Adoption
Perceived Usefulness (PU)	3	PU1. I believe that using a restaurant subscription service allows me to quickly receive relevant information about the restaurant (such as location, distance, menu, etc.). PU 2. I believe that using a restaurant subscription service simplifies the payment process for ordering (e.g., joining as a member and paying the monthly fee online with a credit card). PU 3. Overall, I find that using a restaurant subscription service is beneficial to me.	[29]
Perceived Ease of Use (PEU)	3	PEU 1. I find it easy to use the restaurant subscription service app. PEU 2. I find that using the restaurant subscription service helps me easily understand the menu information. PEU 3. I find that the ordering process through the restaurant subscription service is easy.	[29]
Attitude Toward Use (AUT)	3	AUT 1. Compared to traditional restaurant consumption methods, the restaurant subscription service intrigues me. AUT 2. I find the overall concept of the restaurant subscription service to be appealing. AUT 3. I believe that choosing the restaurant subscription service as a payment method is helpful for frequent purchases.	[29]
Behavior Intention to Use (BIU)	4	BIU 1. If a platform offering a restaurant subscription service is launched, I would be interested in using it. BIU 2. If the restaurant subscription service provides a variety of restaurants and meal options, I would want to continue using it. BIU 3. I would recommend the restaurant subscription service to friends and family when choosing dining options.	[29]
New Environmental Paradigm (NEP)	3	NEP 1. I believe that space and resources on Earth are limited, so food should not be wasted. NEP 2. I believe that humanity should prioritize the responsible use of resources to achieve sustainable development. NEP 3. I believe that everyone should cherish food and possessions and live in harmony with nature.	[39]
Environmental Behavior (EB)	2	EB 1. If the restaurant subscription service offers meals made from surplus food, I might be interested in trying them. EB 2. If the restaurant subscription service provides a platform for surplus food exchange, I might be interested in trying it.	[73]

**Table 3:** Results of factor loading, reliability, and validity.

Items	Factor Loading	Cronbach’s α	CR	AVE
Perceived Usefulness		0.772	0.78	0.55
PU1.	0.64			
PU 2	0.69			
PU 3	0.87			
Perceived Ease of Use		0.877	0.80	0.71
PEU 1	0.87			
PEU 2	0.80			
PEU 3	0.85			
Attitude Toward Use		0.857	0.86	0.68
AUT 1	0.85			
AUT 2	0.88			
AUT 3	0.73			
Behavior Intention to Use		0.925	0.93	0.76
BIU 1	0.87			
BIU 2	0.82			
BIU 3	0.88			
BIU 4	0.92			
New Environmental Paradigm		0.880	0.89	0.73
NEP 1	0.70			
NEP 2	0.94			
NEP 3	0.90			
Environmental Behavior		0.940	0.94	0.89
EB 1	0.96			
EB 1	0.93			

Note: CR: Composite reliability; AVE: Average variance extracted.

**Table 4:** Means, standard deviations, and correlations of constructs.

Construct	Mean	S.D.	1	2	3	4	5	6
1. Perceived Usefulness	4.05	0.72	1					
2. Perceived Ease of Use	4.12	0.72	0.707 **	1				
3. Attitude Toward Use	4.01	0.75	0.663 **	0.670 **	1			
4. Behavior Intention to Use	3.99	0.78	0.693 **	0.673 **	0.790 **	1		
5. New Environmental Paradigm	4.69	0.55	0.150 **	0.170 **	0.265 **	0.190 **	1	
6. Environmental Behavior	3.79	1.08	0.201 **	0.151 **	0.276 **	0.223 **	0.218 **	1

Note: *N* = 369; ** *p* < 0.01.

**Table 5:** The model’s standardized regression weights, *t*-values, R-squared values, and hypotheses.

Path	Standardized Regression Weight	*t*-Value	R-Squared	Hypothesis
Directed effect of the integrative model				
PU → ATU (γ_11_)	0.844	4.722 ***		H1 *
PEU → ATU (γ_12_)	0.288	2.874 **		H2 *
NEP → ATU (γ_13_)	0.161	2.602 **		H3 *
EB → ATU (γ_14_)	0.084	2.904 **	0.562	H4 *
ATU → BIU (β_12_)	0.678	8.157 ***	0.624	H5 *
χ^2^/df = 1.996, GFI = 0.934, AGFI = 0.906, NFI = 0.953, RFI = 0.940, IFI = 0.976, CFI = 0. 976, SRMR = 0.032, RMSEA = 0.052				

Note: *t* > 2.58, ** *p* < 0.01; *t* > 3.29, *** *p* < 0.001; * indicates the hypothesis was supported.

**Table 6:** Results of mediation effect analysis.

		95% Confidence Interval		
Estimate		BC/PC *p*-Value	BC	PC
Indirect effect				
PU → ATU → BIU	0.332	0.002/0.004	0.166~0.562	0.140~0.534
PEU → ATU → BIU	0.187	0.072/0.088	−0.016~0.446	−0.026~0.430
NEP → ATU → BIU	0.070	0.008/0.013	0.021~0.146	0.017~0.138
EB → ATU → BIU	0.079	0.014/0.017	0.012~0.164	0.010~0.161
Direct effect				
PU → BIU	0.352	0.012/0.014	0.088~0.755	0.085~0.748
PEU → BIU	−0.030	0.761/0.792	−0.311~0.178	−0.306~0.183
NEP → BIU	−0.025	0.444/0.396	−0.086~0.041	−0.091~0.038
EB → BIU	−0.044	0.291/0.296	−0.126~0.039	−0.125~0.040
Total effect				
PU → BIU	0.684	0.001/0.001	0.367~1.106	0.354~1.093
PEU → BIU	0.157	0.447/0.364	−0.268~0.447	−0.230~0.474
NEP → BIU	0.045	0.230/0.263	−0.029~0.123	−0.030~0.120
EB → BIU	0.035	0.504/0.479	−0.070~0.127	−0.069~0.129

**Table 7:** Mediating effect of attitude toward use and research hypothesis testing results.

Path	Mediation Effect	Hypothesis
PU → ATU → BIU	Partial mediation	H6 *
PEU → ATU → BIU	No	H7
NEP → ATU → BIU	Full mediation	H8 *
EB → ATU → BIU	Full mediation	H9 *

* indicates the hypothesis was supported.

## Data Availability

The original contributions presented in the study are included in the article, further inquiries can be directed to the corresponding author.
